# Strengthening Workplace Well-Being in Research Animal Facilities

**DOI:** 10.3389/fvets.2020.573106

**Published:** 2020-10-29

**Authors:** Judy Murray, Cassondra Bauer, Nicole Vilminot, Patricia V. Turner

**Affiliations:** ^1^Global Animal Welfare and Training, Charles River, Wilmington, MA, United States; ^2^Laboratory Animal Medicine, Charles River, Ashland, OH, United States; ^3^Veterinary Services, Charles River, Mattawan, MI, United States; ^4^Department of Pathobiology, University of Guelph, Guelph, ON, Canada

**Keywords:** compassion fatigue, resiliency, animal welfare, laboratory animal, mental health

## Abstract

In recent years, there has been an increased recognition of the potential cost of caring on the mental well-being of research animal facility personnel. While this issue is considered a normal consequence of caring for others, these stressors must be acknowledged and managed to ensure that the workplace culture remains positive and that employees are engaged. Factors that can contribute to these feelings in those working with animals in research include compassion and moral stress, issues related to staffing and scheduling of work, insufficient communication in the workplace, and public ambivalence toward the use of animals in science. The first step in developing a program is to survey facility personnel about their concerns, either formally (e.g., using a needs analysis) or informally. Two examples are provided to demonstrate different institutional approaches to assessing personnel needs and developing an internal compassion-resiliency program. The best programs are based on the needs and wants of personnel and these can be cost effective and geared at a grassroots level. Social support in the workplace, for example, through peer counseling, can be a highly effective means of helping personnel to build compassion-resiliency. Addressing mental well-being of research animal facility personnel is an important component of ensuring a positive culture of care in the workplace.

## Introduction

Caring for and working with animals in research environments can bring great joy and pleasure to those working with them; however, it can also result in workplace stress. Promoting a culture of care or well-being within the workplace is the stated goal of many organizations (1). Within laboratory animal science, a culture of care generally refers to promoting good animal welfare practices, ensuring quality of scientific results, promoting transparency and openness about the research process, and ensuring good care and support of employees (2). Well-being of employees working in biomedical research facilities is particularly important for their long-term job satisfaction and retainment (3). Recent surveys have suggested that compassion and workplace stress and fatigue are widespread amongst laboratory animal professionals and others in support roles within the research program, such as IACUC members, security, facility management, trainers, and administrative staff in North America (3–5). Thus, developing programs that support and improve the mental well-being of personnel should be an area of concern and attention for those overseeing research animal facility administration and operations.

This review will cover factors contributing to workplace stress in laboratory animal science, assessing workplace stress in research animal environments, and considerations for developing tools and programs to promote workplace well-being and build resiliency for those working in research animal facilities. The focus is on all personnel and team members who may be working within a research animal environment, including those performing animal care, in-life, and post-life work, as well as those overseeing the research projects, working to maintain facilities, overseeing the physical plant, and managing the animal research compliance office. It is important for administrators and others to note that these work-related stressors are found in all types of animal research environments (for example, universities, government facilities, industry, not-for-profits, hospitals, etc.) and can occur regardless of the species being worked with (for example, rodents only, fish, poultry, etc.) (5). Finally, the modern research animal environment is highly regulated and inspected (6–8), and this paper starts with an assumption of personnel working in an accredited and/or inspected research facility and that the ongoing work with animals is overseen and approved by an appropriately constituted animal care and use committee or oversight body according to national or regional regulations and legislation (7).

## Factors Contributing to Workplace Stress in Animal Research Environments

There are a number of factors that contribute to workplace stress in research animal facilities—both work-related and -unrelated, but this review will only focus on the most significant factors specifically related to the work. Some of these factors are not unique to laboratory animal science; however, the added dimension of working with living, sentient animals creates additional responsibilities and may create additional burdens or stressors. These factors may include moral stress or distress, compassion stress or fatigue, feelings related to a lack of choice or control in the work, insufficient staffing, insufficient communication opportunities at work, and challenges in speaking about their work with others. Each of these areas will be explored further below.

### Moral Stress

The conflicting feelings that laboratory animal science professionals and others working in research with animals may experience from time to time related to their work can be due to moral stress. These same feelings are also frequently reported by those working in nursing, human medicine, veterinary medicine, palliative care, and social work (9–13). Moral stress refers to having to act in a way that is different from what someone feels is ethically correct. The reasons underlying why this occurs in the different “caring professions” are different; however, when feelings of distress happen repeatedly over time without an opportunity to redress issues or properly recharge, it can lead to occupational “burn-out” (14). Animal euthanasia is an area of moral stress in research settings. There is genuine acceptance by most laboratory animal professionals about the importance of working with animals in science to enhance fundamental knowledge as well as to make advances in biomedical research science, particularly within a 3Rs framework (15). Most people working in research care deeply for the animals they work with, regardless of the species (5). However, euthanasia of a cohort of animals is often necessary at the end of an experiment to gather additional information about physiologic and pathologic processes from tissue or other samples. Despite understanding the need for this action, it can be challenging to conduct euthanasia of animals and the action may result in feelings of grief and moral stress. These feelings can be compounded if euthanasia is required at regular intervals, different endpoints for animals might be possible but are never discussed or considered (e.g., rehoming or adoption of animals), there is poor communication about the task or a lack of choice or opportunity to discuss ongoing feelings about the work, and individuals do not have the tools needed to be resilient. Moral sensitivity is important because it emphasizes the role of ethics and social values when working with research animals (16). However, individuals working with animals must be allowed to discuss the ethical implications of their work, including the constraints under which the work has been determined to be acceptable by the institutional animal ethics committee.

### Compassion Stress and Fatigue

Compassion refers to bearing the suffering of others (16). In research settings, and following ethics committee approval, researchers may induce disease or other conditions in animals or administer treatments that intentionally induce suffering, distress or pain as a condition of the animal model being studied. Periods of discomfort or distress of animals are limited to the extent possible by means of anesthesia, analgesia, and humane endpoints or interventions [for examples, see (17–19)]. Compassion stress is the forerunner to compassion fatigue and is thought of as the emotional burden following providing care to relieve suffering of others—either human or animal (16, 20). For those who love animals, there is a cost to providing good care in research settings, which requires constant empathy and emotional investment in the animals worked with (16). Compassion is not unlimited and also can be consumed by events and activities that are indirectly experienced or witnessed, also known as secondary trauma (16). When unattended to, compassion stress can build over time to become compassion fatigue. When this occurs within the context of a demanding work environment, it can lead to burn-out; however, it is important to note that burn-out in the workplace is an occupational hazard (14) and may occur in the absence of compassion fatigue. While compassion stress and fatigue have been recognized as occupational hazards in research animal settings for over two decades, it is only recently that mental well-being of workers has been of interest and institutions have become aware of the need to provide support for their employees.

### Staffing and Scheduling Factors

It has long been known that the quality and number of animal care and veterinary professional staff are critical factors in determining the overall quality of an animal research program. Balanced with this is the notion that personnel costs can represent up to 65% of a program's costs (21). The fluctuating and uncertain nature of scientific funding in today's academic environments and the variability of sponsored studies in private industry coupled with increasingly lean operational strategies and normal attrition rates and turnover of personnel can mean that it is difficult to ever fully staff research animal facilities. This can lead personnel to experience feelings of frustration and even despair at being unable to complete their daily work, in addition to feelings of guilt when taking scheduled breaks, lunches, and vacations. In addition, research animal environments can be highly scheduled offering little perceived control and choice for workers, factors that are known to be important for improving employee performance and mood (22). Animals must be fed, cleaned, and observed at certain times, treatments and timing of sample collections are often highly proscribed, mandatory overtime (with pay) is often necessary and may be required at short notice, and the risk of an adverse outcome following an error with living animals is high. All or any combination of these factors can create job strain in those working in research animal environments as well as feelings of effort-reward imbalance (23). Effort-reward imbalance is characterized by a recurring lack of reciprocity between the efforts expended at work and the rewards—both direct and indirect—received in return (23). In similar fields with similar demands, i.e., human health care, high levels of occupational stress and effort-reward imbalances have been noted with job dissatisfaction rates reported of up to 1 in every 4 workers (24, 25). In addition to mental health effects, chronic work stress can contribute to increased risks for coronary heart disease (23). Those working in research animal facilities have not been specifically studied for rates of job dissatisfaction, but lab animal professionals commonly report feelings of stress, high workload, and burn-out (5).

### Factors Related to Inadequate Communication

Good communication is essential in any workplace, but it is particularly critical when the care and lives of research animals are at stake. Those working with and caring for animals are often strongly attached to the animals in their care and invested in their well-being (26). Delays, real or apparent, in animal care, treatments or other procedures, such as weaning or endpoint decision-making, can lead to personnel distress and feelings of helplessness, as individuals become worried about the future welfare state or condition of vulnerable animals. In these situations, there may not be intentional exclusion of stakeholders concerned with animal well-being, in that veterinary and research staff may communicate about and move forward with next steps in an experiment, while neglecting to feed information back to those working directly with animals.

Similarly, an inability for those working in research animal facilities to speak openly about their questions and concerns related to animals or research, because of a lack of workplace openness, can lead to workplace stress (3, 27). In human health care fields such as nursing, a perceived lack of opportunity to discuss concerns is an important source of job stress (28). Thus, modeling good communication and encouraging openness in discussing research animal concerns are important considerations for long-term retention and satisfaction of those working in research environments.

### Public Discomfort With Research Animal Experiments

In Western society, the public has an uneasy and mixed relationship to the use of animals in research (29). On the one hand, safe and efficacious treatments, vaccines and cures are demanded; however, there is an unwillingness to openly discuss exactly how these needs can be met. A lack of overwhelming support for animal research by society at large (30) can create workplace stress in research animal workers. Self-esteem and value are commonly tied to the nature of one's work (31, 32) and the inability to speak about one's work to peers, friends or family members can contribute to feelings of discomfort and shame in research animal workers (4). Regularly communicating about the importance of the research being conducted can help to increase fluency in employees about the science. Additionally, teaching employees how to speak about their work and providing opportunities for them to share aspects of their work with families and friends can result in the feeling of removing an enormous burden from individuals (4, 5).

## Assessing Workplace Well-Being in Research Animal Facilities

Given the increasing recognition of the importance of workplace well-being and that there are known factors for stress and distress in research animal environments, conducting some form of workplace needs assessment may be beneficial to identify gaps between the present and desired state in a facility. An assessment may be informal or formal, qualitative, quantitative, or use a mixture of methods (33) and the approach used may depend on the resources available at the facility. Whatever means are used, personnel should have an opportunity to express their honest feelings in a safe environment. Often, this is best accomplished by making use of a facilitator with no direct relationship to any of the employees. No matter how good the relationship with the direct supervisor, manager or administrator, it can be difficult for employees to be completely candid in their comments about challenges in their work environment.

It is beyond the scope of this paper to delve fully into how a formal workplace needs analysis is conducted and the reader is referred to other discussions on this topic (33, 34). Briefly, a needs analysis identifies the desired outcome or state for the workplace environment, describes the current situation (for example, via surveys, focus groups or interviews), describes the gaps between the two states and the causes for them, and identifies possible solutions for bridging the gaps (i.e., generation of a prioritized action plan) (34). An example of one real-life approach to a largescale facility-wide needs analysis at a large research animal facility is provided in Box 1.

Box 1 **Case example for conducting a formal needs assessment at a large research animal facility.**

The need to initiate a program to help employees experiencing compassion fatigue was identified at a preclinical safety facility with about 1,000 employees. Concerns were expressed to site management from employees regarding their feelings during animal studies, as well as other aspects of scheduling that were contributing to job stress. The site had recently changed ownership, but these emotions were longstanding, stretching back at least 4–5 years. Senior management at the site discussed the issues brought forward and based on their concerns to improve institutional culture, determined to create a program to help support employees and prevent them from developing burn-out. Because many of the issues seemed to be longstanding, the site elected to pursue a formal needs assessment process.

As part of the needs assessment, an anonymous internal survey was developed and distributed to employees at the facility. The survey assessed employee understanding of compassion fatigue, coping mechanisms utilized, feelings about administrative support for employees, and asked respondents to rank various ideas regarding what the program should first address. The survey return rate was 14%, representing individuals from a wide variety of tenure and job skills, including those who worked with animals directly and those who did not. Additionally, an external compassion fatigue consultant was engaged to assist with assessment of the needs of the facility.

Once on site, the external consultant provided a short presentation about compassion fatigue that was open to all employees and that was repeated several times to accommodate schedules for all interested personnel. Following this, individual and group interviews were scheduled with the consultant in a private office. Employees met with the consultant to discuss their confidential concerns about work-related issues and perceived stressors as well as providing information about how they coped with job stresses. Following the consultant's visit, a report was generated and shared with site management. The report detailed aspects of workplace stress with suggestions for opportunities that would alleviate some of the stressors, as well as pinpointing individuals who were interested in helping to build an employee support program. Some of the items identified by the consultant in the report were surprising, others were known, and many had already been addressed, and yet people still referred to these past issues as sources of stress, likely because of a perceived lack of opportunity to fully discuss their concerns at the time that events occurred.

**Figure 1:**
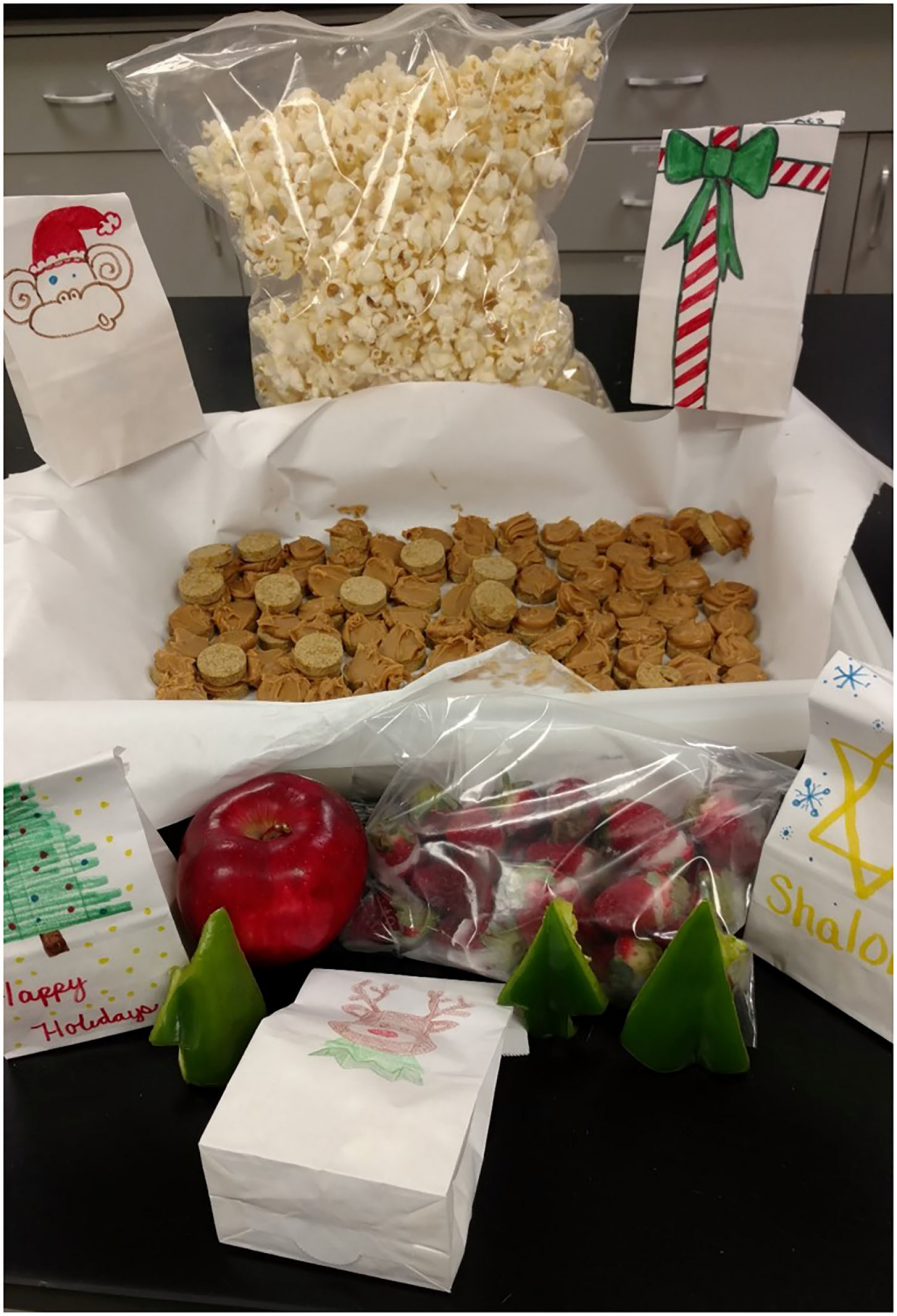
An example of the holiday treats for animals program. Facility personnel come together to create and then distribute healthy treats for animals in the facility. In this example, there is air-popped popcorn for rodents, green pepper Christmas trees for rabbits, apple rings for pigs, peanut butter cookies for dogs, and Santa hats (strawberries and yogurt) and treat bags for primates.

Following the consultant's visit, a second internal anonymous survey was distributed to the employees. Respondents indicated that the information presented and the opportunity to speak with the external consultant about compassion fatigue concerns were valuable. Subsequently, additional personnel were added to areas identified to have high levels of work-related stress, allowing for fewer hours engaged in challenging tasks and increased rotation through different tasks to add variety. A small compassion-resiliency committee was formed after the consultant's visit. This group solicited opinions and ideas from different business groups at the site, which were subsequently prioritized and presented to management for approval and resource allocation. Where possible, events were combined with previously scheduled staff appreciation events to maximize impact, and interests and needs of different groups were taken into account when planning activities. One example is the concept of holiday treats for animals, in which employees are given time during their working day to come together to create and distribute special themed treats for the animals in the facility (Figure 1). The compassion fatigue program has grown slowly over time, gaining traction and interest among many employees as it develops.

A second real-life example of a more informal approach to a needs assessment for a large research animal facility is provided in Box 2.

Box 2 **Case example for conducting an informal needs assessment at a large research animal facility.**

**Figure 2:**
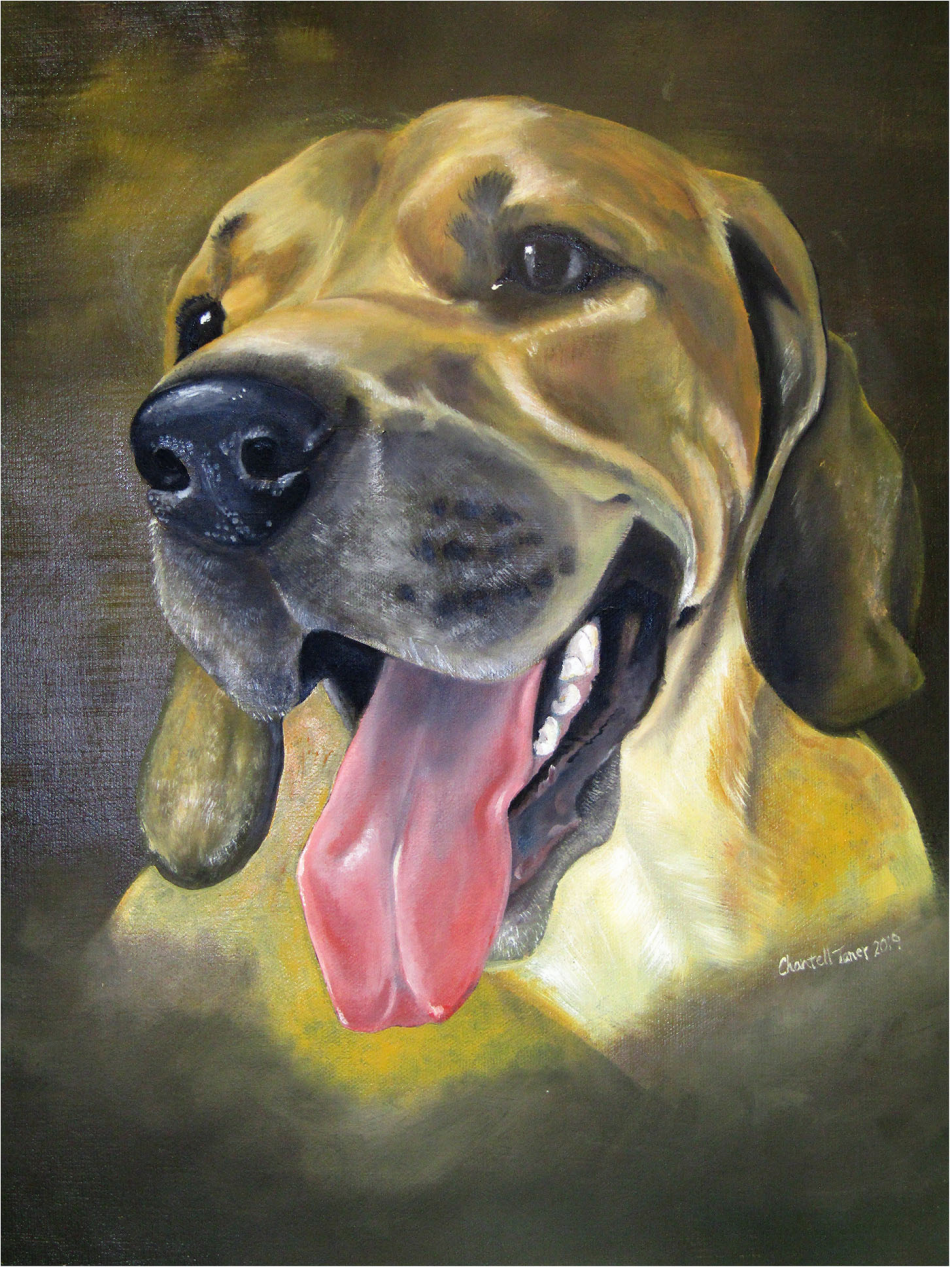
An example of a painting from an Art of Compassion program. Courtesy of T. Custard.

The years following the 2008 economic decline in the U.S. were difficult ones for this preclinical safety facility. Budget cuts had resulted in numerous employee lay-offs, personnel morale was at a low point, and spending for non-essential projects was discouraged. Serendipitously, a small victory was achieved when funding was donated for a tribute garden for the animals. The concept of a tribute garden was approved by site management and personnel were given paid time to develop the project with a lasting, significant impact for staff. The garden marked the start of the entire compassion-resiliency program at the facility. Publicly celebrating the human-animal bond wasn't something that employees at the site were accustomed to, but once staff were encouraged to open up about their relationships with the animals they worked with, the organization saw increased openness and engagement of employees That openness empowered the creation of several additional programs, such as an adoption program and an “art of compassion” program. This latter program allows personnel to request a portrait of an animal that they have developed a special bond with (Figure 2). Employees who are accomplished artists volunteer to draw or paint the animal as a keepsake for the requesting technician. Whereas, the site used to discourage and deny the bonds that are formed with animals are being cared for, they now celebrate and encourage them. While the artwork directly impacts those requesting it, the site also discovered that the success of the program reaches far beyond the vivarium walls. By displaying the artwork around the facility, an important and lasting celebration of the human-animal bond can be made with all employees as well as site visitors. When people see a visual representation of the bonds between employees and the animals, it fosters an environment in which everyone feels valued for what they do.

The compassion-resiliency program at the facility is ever-evolving. Initially those helping to organize the program were excited simply to be able to implement projects that resonated well with employees, but they've since evolved into thinking about the program through a long-term lens. While personnel have been recruited as ambassadors within the facility to help develop projects that will keep staff engaged, the program also focuses on management awareness and employee education. The program organizers are trying to validate the emotions that employees encounter when working closely with not only animals, but each other. By doing this, they hope to foster an environment in which people will be encouraged to express their own ideas of what they need. The ultimate goal of the program is to positively impact the overall culture of the facility.

These two examples are provided to demonstrate that successful outcomes can be achieved in research facilities using both formal and informal approaches to needs assessments. In both cases, intentional efforts were made to engage personnel in discussions about how the workplace could be improved. Employees were given a large measure of control over how they chose to address challenges that had been identified in the workplace, contributing to buy-in and sustainability of programs.

It is important to note that compassion fatigue and moral distress are often not a constant state for personnel in research animal facilities. In resilient individuals, including those with more choice and control over their workday, these feelings may never or rarely be experienced or feelings may come and go from time to time, depending on other external factors that impinge on employees' lives. Secondary stressors, including strained relationships with partners or children, fears and concerns arising from personal and family health issues, financial health concerns, unrelated anxiety or mood disorders, etc., may exacerbate work stress (35). Working closely with research animals brings many joys for those who are attracted to being with animals, often called compassion satisfaction, because of a strong human-animal bond that may develop (26). The balance between the joy and challenges that may be experienced in research animal work is often referred to as the professional quality of life or ProQUAL (36). Institutions should strive to ensure that programs and supports are in place such that the professional quality of life experienced by laboratory animal professionals is generally positive. An online ProQUAL survey is available and can be adapted for research animal facility use with a few edits (37). This could also be used as a less formal means of gauging mental well-being of employees.

## Tools and Programs to Promote Workplace Well-Being in Research Animal Facilities

As one more closely examines the causes of workplace stress in research animal facilities, there is clear evidence that resiliency plays an adaptive and protective role, as both a coping mechanism and a way to increase compassion satisfaction associated with the work. Resilience refers to one's ability to cope with and bounce back from stress and adversity. Resilience is not static, there may be times when one's capacity to deal with challenges ebbs and flows, but resiliency can be enhanced through intentional practice and adaptation to new situations. As mentioned, compassion stress is a component of working with animals in science. However, multiple studies have demonstrated that individuals and organizations that incorporate resiliency building into their practices promote both human and animal well-being (3, 4). Supporting implementation of the 3Rs (replacement, reduction, and refinement), empowering people to be creative problem solvers, providing opportunities to report questions or concerns, and expecting accountability at all levels, creates a work environment that values people, animals, and science (2). Refinements in handling techniques or procedures to minimize stress, providing food resources and other enrichment, and opportunities to interact closely with animals are shown to have a positive effect, increasing compassion satisfaction and professional quality of life (4). All these activities help employees adapt and overcome challenges discussed previously, building their resiliency in the process.

A Compassion Fatigue Resiliency (CFR) model was recently developed by a group of researchers (38) as a tool to determine the level of risk for individuals to experience compassion fatigue or to develop levels of resilience that subsequently reduced the impact of compassion fatigue. The model uses 12 variables as predictors of CFR related to empathy, secondary traumatic stressors, and compassion fatigue resilience. These and other authors have indicated that building resilience depends largely on nurturing positive practices of self-care, developing some degree of detachment or respite from work-related stresses, enhancing a sense of satisfaction or fulfillment, and developing strong social supports (4, 38–40). This concept of resilience and how it can be used to increase employee resiliency is fundamental for strengthening workplace well-being in research animal facilities.

Coping with challenges in the research animal environment must be approached in a multi-pronged fashion, with changes aimed at both individual and institutional levels. As individuals, building compassion-resilience is focused on behaviors, thoughts, and attitudes that support physical and mental health (41). Sleep, nutrition, exercise, and mindfulness practices (e.g., yoga, journaling, meditation) help recharge energy and provide a break from work stresses. A recent survey has found that all of these self-care methods are seen as valuable stress relievers (5). Helping others, through volunteering or supporting a friend or family member in need, helps people to find purpose and fulfillment. Embracing a “growth mindset” and learning to reframe challenges and setbacks as opportunities to learn and grow can help individuals to adapt and thrive when facing adversity (41). Social connection is one of the best ways to build resiliency. While connecting with others outside of the workplace is helpful, developing a support system within family and friends also reduces the risk of social isolation and is reported to decrease feelings of compassion fatigue (3). Connecting with co-workers offers another form of social support and reminds employees that they are not alone, are heard, and helps to validate their feelings (4, 17–19). A recent study found that increased social support for research animal workers was related to higher compassion satisfaction, reduced perceptions of animal stress or pain, and improved human-animal interactions (4).

At an organizational level, the first step in addressing workplace challenges within research animal facilities is acknowledging that compassion stress and fatigue are normal aspects of caring for others and that this occurs in many occupations. Institutions want people who are compassionate and empathetic when working with animals, dedicated to providing the best care possible. There is abundant research on the importance for organizations to provide education and training on recognition of compassion stress and fatigue, and to establish emotional support programs and resources for personnel (3, 4, 42). Well-being and resiliency education should start early after an employee has been hired to work in a research animal facility. People entering the field are at higher risk of anxiety incurred from animal use, particularly in those with <2 years of experience (43). Organizations can increase feelings of satisfaction and fulfillment for employees by reminding employees of the importance of their individual contributions to the advancement of science and improvement of societal quality of life. Assuring that personnel are well-trained can reduce stress and increase confidence in employees' technical skills, increasing job satisfaction, and compassion satisfaction.

Acknowledging the value of the human-animal bond, which brings both compassion stress as well as compassion satisfaction, and encouraging open dialogue regarding animal research, euthanasia and the accompanying moral stresses can help to build resiliency. Providing choice for personnel to participate in euthanasia events for animals they have cared for is also an important means of addressing workplace stress (4). Another important area for reducing workplace stress is enhancing communications so that personnel are aware of the work being done with animals and are apprised of changes and updates in experimental plans can provide significant relief from workplace stress. When animals are no longer needed for research projects, a priority should be placed on retiring, rehoming or adopting these animals out to a forever home, when possible. While this may require additional effort and resources to prepare animals for their new life outside the research facility (e.g., IACUC review and approval, vaccinations, neutering, etc.), personnel are generally excited to support the process. Developing internal recognition programs for those who exceed expectations in their daily care of animals or who develop new 3Rs approaches for working with or replacing animals in science is also important as it encourages employees to do their best at work each day. Finally, it is imperative that institutions foster opportunities for connection, engagement and social support as these are foundational to building resilience in research animal facility employees. Nurturing ways for personnel to find satisfaction in their work contributes to enhanced mental wellness and a deep appreciation and care for the animals they work with.

With the change in workforce demographics, early career employees are increasingly focused on personal fulfillment, engagement, support, and well-being in the workplace (43, 44). While compassion stress and compassion fatigue are not new to this field, awareness of their impact on animal welfare, the welfare of the employee, their teams and the science they support is gaining attention. One model for proactively addressing the cost of caring in research animal facilities is to develop a compassion-resiliency building program that provides at its core strong social supports, some level of choice or control for the employee, and the means to respect the human-animal bonds that enhance compassion satisfaction and resilience. As every facility is different, with a different work focus, set of species worked with, culture, and employee needs, each program should be developed by using grassroots methods and listening to employee needs and wants. Examples of two possible approaches for this were provided in Box 1 and 2, one using a formal needs assessment and the other demonstrating organic growth from one idea that was meaningful to employees. A summary of areas and tools that might be considered as part of an institutional compassion-resiliency building program is found in Table 1.

**Table 1:** Examples of activities and programs that support resiliency in research animal facilities.

**Category**	**Activities and programs supporting resiliency**
Social supports	Peer counseling
	Staff engagement activities
	Invitations for researchers to discuss their work with facility personnel, e.g., “meet the researcher” lunches
	Ongoing communications about animal experiments with in-life personnel
Acknowledging human-animal bond	Animal naming
	Providing tributes to animals
	Scheduling time for human-animal interactions, e.g., animal grooming, dog walking, gentling
	Providing choice for animal euthanasia events
Regular assessment of animal behavior and welfare	Comprehensive animal behavioral management programs
	Implementation of animal welfare assessment programs for all species
Strong 3Rs programs	Advocacy for animal replacements and refinements
	Animal retirement, adoption and rehoming programs
	Internal 3Rs awards
Promoting self-care	In-house fitness facilities or reimbursement for fitness programs
	Wellness programs, e.g., nutrition support, mindfulness training
	Yoga and meditation classes
Learning and development	Regular CE regarding 3Rs and animal welfare
	Compassion fatigue and resiliency building training for management and personnel
	Ongoing technical skills development and assessment for proficiency for animal work
Personnel recognition programs	Animal welfare specific awards
	Institutional participation in Biomedical Research Awareness Day (BRAD)
Service to the community	Public outreach regarding biomedical research

Having senior leadership and human resources support for this type of program is essential as there are operational and financial resources needed as well as employee needs and possible synergies with other wellness programs at the local level. Some awareness training may be needed for institutional administrators and researchers who are far removed from animal work. While many institutions subscribe to an Employee Assistance Plan service that provides confidential short-term support for employees experiencing personal difficulties, individuals working within these services often do not have knowledge or experience of the various challenges associated with working with animals in research. A more permanent and ongoing, low cost support system may be needed. Within organizations, there are often a few sympathetic individuals whom others feel comfortable talking with or seeking advice from. These are empathetic employees who are often peers of those working directly with animals, who understand the nature of the work and the emotions that accompany it. Engaging the support of these individuals as peer counselors in the workplace can create opportunities for employees to informally talk about their work, building sustainable social support locally (4, 44).

Having a compassion-resiliency program in place across a research animal institution supports a healthy work environment, reflects values and ethics related to a culture of care, and demonstrates institutional commitment to employee engagement and mental well-being.

## Discussion

Workplace stress has been a topic of discussion in research animal facilities for decades, yet remains in the shadows for many working inside and outside the vivarium (27). This lack of awareness can leave caring and compassionate people feeling alone, anxious, and unsupported. Bringing the discussion about work stressors and mental well-being of employees into the open requires addressing concerns of management, human resources, legal and other stakeholders regarding possible effects on their workforce. Focusing on positive outcomes of resiliency building is recommended rather than negative attributes such as compassion or moral stress. Efforts at building awareness within employees is also needed. There can be workplace stigma associated with speaking about mental health, which further emphasizes the need for educating supervisors, managers, and administrators. Without their support, work cultures will not easily change. Workplaces that promote mental well-being see reductions in absenteeism and increased productivity (2, 14).

Sustaining a compassion-resiliency building program over time requires commitment at the organizational as well as local level. The program should also not just focus on promoting self-care and resilience but acknowledge and address the other risk factors that impact human and animal well-being. Addressing staffing levels, workload, mandatory overtime, training, available resources, choice or control over daily tasks and schedules, etc., are all areas the organization can look at to address sources of workplace stress. Creating infrastructure for the program at the outset will ensure that it is a sustainable model that grows organically, meets personnel needs most effectively, and provides resources for people in crisis that Employee Assistance programs are not always equipped to handle.

## Conclusion

Workplace stress can be a significant issue for those working in research animal facilities and is a normal consequence for individuals working in a caring profession. The current emphasis on mental well-being in the workplace provides an ideal opportunity for institutions to develop programs of support and to critically appraise expectations for those providing care and working with animals in science. An important means of addressing this issue is to develop a grassroots compassion-resiliency program that has at its core an emphasis on social support for employees in the workplace. Ensuring that personnel are well-supported and resilient contributes to a positive culture of care in the workplace, increased job satisfaction and personnel retention, and enhanced care and well-being of animals.

## Author Contributions

All authors contributed to conception and design of this paper. JM and PT developed the paper outline and all authors contributed to the first draft of the manuscript. All authors revised the manuscript, and read and approved the submitted version.

## Conflict of Interest

The authors declare that the manuscript was prepared in the absence of any commercial or financial relationships that could be construed as a potential conflict of interest.
